# Modification of the therapist’s facial expressions using virtual reality technology during the treatment of social anxiety disorder: a case series

**DOI:** 10.3389/fpsyg.2023.1030050

**Published:** 2023-05-15

**Authors:** Toshiro Horigome, Shigeo Yoshida, Tomohiro Tanikawa, Masaru Mimura, Taishiro Kishimoto

**Affiliations:** ^1^Department of Neuropsychiatry, Keio University School of Medicine, Tokyo, Japan; ^2^Department of Psychiatry, Shonan Keiiku Hospital, Kanagawa, Japan; ^3^Research Center for Advanced Science and Technology, The University of Tokyo, Tokyo, Japan; ^4^OMRON SINIC X Corporation, Tokyo, Japan; ^5^Next Generation Artificial Intelligence Research Center, The University of Tokyo, Tokyo, Japan; ^6^Hills Joint Research Laboratory for Future Preventive Medicine and Wellness, Keio University School of Medicine, Tokyo, Japan; ^7^Donald and Barbara Zucker School of Medicine at Hofstra/Northwell, New York, NY, United States

**Keywords:** social anxiety disorder, exposure therapy, facial expressions, virtual reality, fear of negative evaluation

## Abstract

Exposure therapy is a mainstream of treatment for social anxiety disorder (SAD). However, effort and time are required to recreate interpersonal situations that produce moderate anxiety. On the other hand, virtual reality exposure therapy can easily control anxiety-inducing conditions and allow for graduated exposure. However, artificial intelligence and animations that speak as naturally as actual humans are not yet practical, adding to the limitations of these treatments. The authors propose the use of a virtual reality technology that can transform facial expressions into smiling or sad faces in real time and display them on a monitor, potentially solving the above-mentioned problems associated with virtual reality animations. This feasibility study was conducted to determine whether this system can be safely applied to the treatment of SAD patients. A total of four SAD patients received 16 exposure therapy sessions led by an experienced therapist over a monitor; throughout the sessions, the facial expressions of the therapist were modified using software to display expressions ranging from smiling to sad on the monitor that was being viewed by the patient. Client satisfaction, treatment alliance, and symptom assessments were then conducted. Although one patient dropped out of the study, treatment satisfaction and treatment alliance were scored high in all the cases. In two of the four cases, the improvement in symptoms was sustained over time. Exposure therapy in which the interviewer’s facial expressions are modified to induce appropriate levels of anxiety in the patient can be safely used for the treatment of SAD patients and may be effective for some patients.

## Introduction

Social anxiety disorder (SAD) is characterized by a strong fear of situations involving other people’s attention. Its prevalence varies by geographical region; for example, the 12 month prevalence rate in the United States is 6.8%, making it the third most common mental disorder (Kessler et al., 2005). After disease onset, social life becomes disturbed as the patient begins to avoid interpersonal situations, resulting in high social costs (Patel et al., 2002). Since less than 25% of patients achieve remission after 2 years of drug therapy and only 35% achieve remission after 10 years (Yonkers et al., 2001; Keller, 2006), the disease often follows a chronic course. Exposure therapy has been shown to be effective as a major treatment method other than pharmacotherapy (Heimberg et al., 1985; Ponniah and Hollon, 2008). However, when exposure therapy is conducted for SAD patients, a great deal of effort and time are required to recreate interpersonal situations capable of causing adequate anxiety (e.g., exposure to public speaking requires gathering people together and controlling their reactions).

The development of virtual reality (VR) exposure therapy (VRET) for anxiety disorders has recently been attempted (Mishkind et al., 2017). VR enables the artificial creation of various situations and can more easily control the conditions that induce anxiety, compared with *in-vivo* exposure therapy, making gradual exposure possible. Studies have also been conducted to examine the effects of VRET for SAD by reproducing speaking and eating situations. The effect of VRET on SAD has been confirmed in several meta-analysis. In comparison with psychotherapy using *in-vivo* exposure, the results showed non-inferiority of efficacy at post-treatment time points (Chesham et al., 2018; Horigome et al., 2020). However, it is unclear whether VRET or *in-vivo* exposure has superior long-term effects, as there are few reports comparing the effects of two groups longitudinally. While some meta-analysis reported that VRET is also non-inferior to psychotherapy with *in-vivo* exposure in terms of long-term efficacy (Kampmann et al., 2016), a meta-analysis that included more trials indicated that it may be inferior to *in-vivo* exposure in the long term (Horigome et al., 2020).

To increase the effectiveness of VRET, the need to make the sociocultural context of the VR scenario resemble the environment in which the subject is located has been noted (Emmelkamp and Meyerbröker, 2021). However, even with these efforts, it is difficult to eliminate the context of using VR. The lifelikeness of VR animations used in previous studies was insufficient, and the available conversational responses were limited. Artificial intelligence and animations that speak as naturally as actual humans are not yet practical. Therefore, we thought that controlling the facial expressions of actual therapists engaged in natural conversations with their patients might be effective for eliciting anxiety in a step-by-step manner. Functional-brain imaging studies suggest that patients with SAD show more amygdala activation than healthy controls when perceiving negative facial expressions and that amygdala activity is correlated with the severity of SAD (Stein et al., 2002; Straube et al., 2005; Phan et al., 2006). Therefore, it may be possible to control the anxiety level of SAD patients by displaying real-time modifications of the therapist’s facial expressions. We have developed software that can transform facial expressions captured by a 3-dimensional camera into smiling or sad faces in real time and display them on a monitor. We hope that in the future, this system can be used to treat patients with SAD, enabling interviews to be conducted with appropriate control of the patient’s anxiety, thereby improving the effectiveness of regular psychotherapy, the persistence of treatment effects, and the rate of treatment continuation. The present feasibility study was performed to determine whether this system can be safely used to treat SAD patients. This study was an exploratory investigation that was not performed based on any rigorous scientific or therapeutic guidelines and was also not intended to investigate the effects of the intervention.

## Methods

Subjects who met the DSM-5 diagnostic criteria for SAD were included. Subjects with pre-existing medical conditions such as bipolar disorders, schizophrenia spectrum disorders, or substance-related disorders, those with imminent suicidal ideation, and those who had received other structured psychotherapy within 12 months were excluded. Recruitment of the participants was conducted through referrals from their primary psychiatrists.

**Figure 1:**
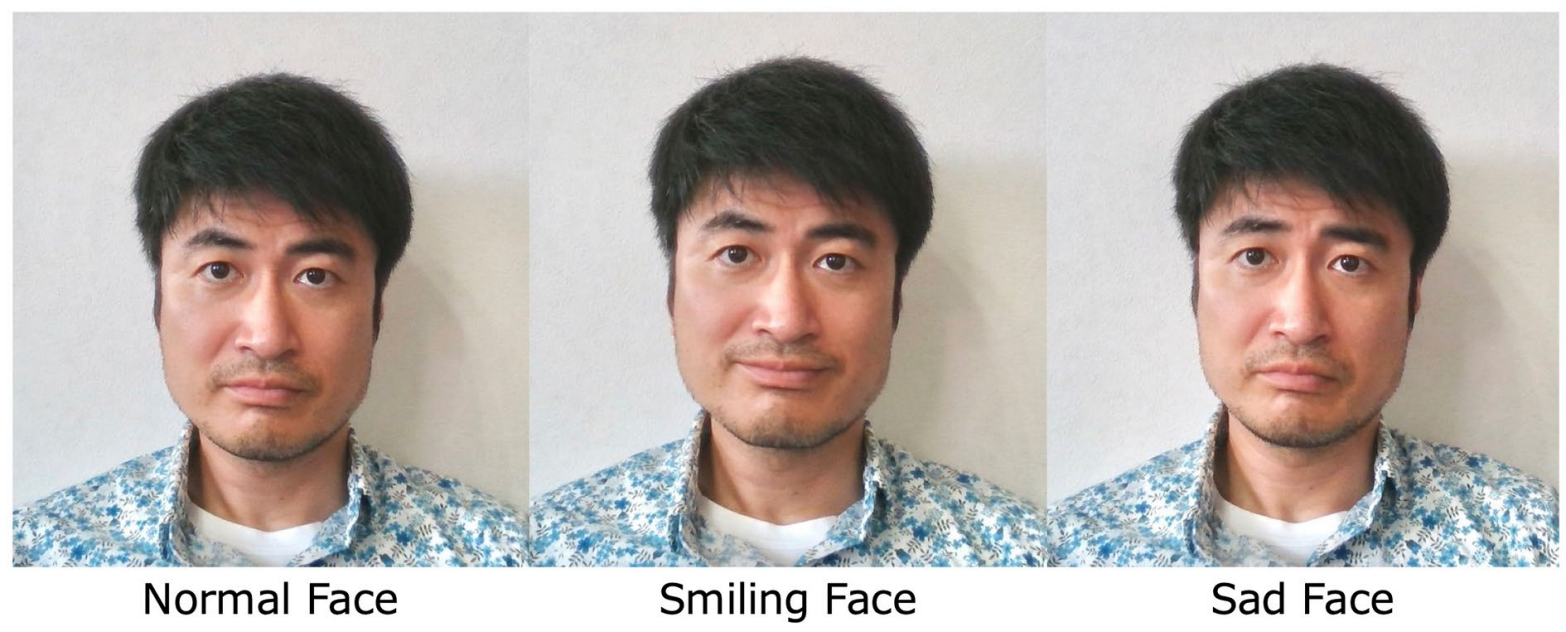
Facial expression alterations created using our software.

In this study, the hospital and participants’ houses were connected via a web conferencing system for conducting patient interviews over a computer monitor. The interviewer’s face was automatically captured by a 3-dimensional camera (BlasterX Senz3D), and the interviewer’s facial expressions were virtually transformed into smiles or sad faces in real time by the image processing technique used in the authors’ previous work (Suzuki et al., 2017). This technique was used to adjust the intensity of the participants’ anxiety by adjusting the interviewer’s facial expression appearing on the computer monitor (Figure 1). The same therapist conducted all the exposure therapy sessions in all the cases, and 16 sessions were scheduled once a week for 40 min, in principle. Conversations were not structured, and the conversational themes were set freely during each session. In the first session, participants received an explanation of this study, including its purpose, duration, frequency, and the significance of modifying the interviewer’s facial expressions. At the beginning of each session and occasionally during the session, the participants were asked to report their level of anxiety on a scale of 1 to 10, and any changes in their anxiety level were shared with the interviewer. When the participant became less anxious with a particular facial expression, participants and interviewers discussed whether they should change the facial expressions to ones that elicit stronger anxiety.

No restrictions were placed on usual outpatient care, such as medication, which was performed in parallel with the study. The measured outcomes included the Working Alliance Inventory-Short Form (WAI-SF) (Tracey and Kokotovic, 1989; Takasaki et al., 2020) at weeks 1, 8, and 16, the Client Satisfaction Questionnaire (CSQ-8) (Nguyen et al., 1983) at weeks 8 and 16, and the Liebowitz Social Anxiety Scale (LSAS) (Liebowitz, 1987) and the Fear of Negative Evaluation Scale (FNE) (Watson and Friend, 1969) at weeks 0, 8, 16, and 24, respectively; the Japanese versions of these measures were used.

The ethics committee of Shonan Keiiku Hospital approved the study, and all the participants provided written informed consent. The study was registered with the University Hospital Medical Information Network (UMIN 000033878).

## Results

**Figure 2:**
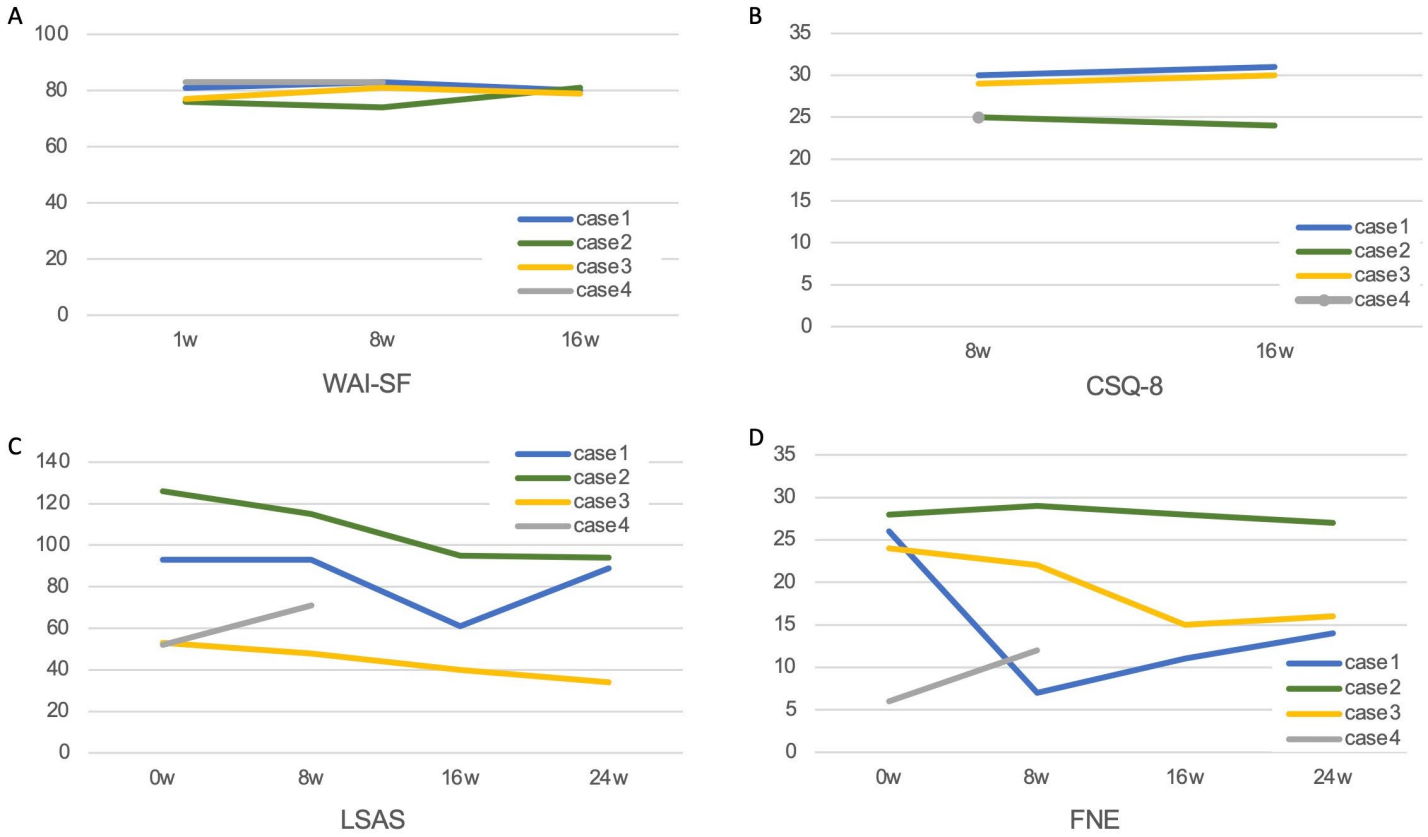
Changes in measures. **(A)** WAI-SF (Working Alliance Inventory-Short Form), **(B)** CSQ-9 (Client satisfaction questionnaire), **(C)** LSAS (Liebowitz Social Anxiety Scale), and **(D)** FNE (Fear of Negative Evaluation Scale).

A total of four subjects (1 male, 3 females, mean age, 31.0 ± 9.9 years; mean duration of illness, 17.3 ± 6.8 years) participated in the study. All the participants were being treated with Selective Serotonin Reuptake Inhibitors, and their medications were not changed during the study period. The measure results for each case are shown in Table 1 and Figure 2. The WAI-SF was 79.3 ± 3.3 at week 1, 80.3 ± 4.3 at week 8, and 80.0 ± 1.0 at week 16. The CSQ-8 was 27.3 ± 2.6 at week 8 and 28.3 ± 3.8 at week 16. In all the cases, patients whose primary psychiatrist was the interviewer in this study were recruited; thus, the interviewer in the sessions and the attending psychiatrist were the same person.

**Table 1:** Changes in measures.

		Baseline	8 w	16 w	24 w
Case 1	WAI-SF	81	83	80	
	CSQ-8		30	31	
	LSAS	93	93	61	89
	FNE	26	7	11	14
Case 2	WAI-SF	76	74	81	
	CSQ-8		25	24	
	LSAS	126	115	95	94
	FNE	28	29	28	27
Case 3	WAI-SF	77	81	79	
	CSQ-8		29	30	
	LSAS	53	48	40	34
	FNE	24	22	15	16
Case 4	WAI-SF	83	83		
	CSQ-8		25		
	LSAS	52	71		
	FNE	6	12		

WAI-SF, working alliance inventory-short form; CSQ-8, client satisfaction questionnaire; LSAS, Liebowitz social anxiety scale; FNE, fear of negative evaluation scale.

One of the patients (Case 4) was affected by a natural disaster and dropped out because the environmental changes made it difficult to continue the study. Therefore, the study outcomes for three cases are presented below.

### Case 1

Case 1 was a 25 year-old male with a 13-year illness duration. He had been unemployed for 3 months and had been living confined to his home. He was aware of his difficulty in maintaining free conversation, and the first half of the sessions were spent practicing free conversation. The contents of the conversation were set to trivial themes, such as favorite foods and taste in clothing. During the conversation, ideas on how to make the free conversation livelier were exchanged. When he experienced a change in the therapist’s facial expression, he reported being more afraid of a smiling face than of a normal face, and he expressed an even stronger fear of a sad face. Nevertheless, after the third session of exposure to sad faces was performed, his fear gradually decreased, and from the fifth session, the sad face began to be used most of the time. From that point on, he began to talk about increasing the things he could do between sessions, and he discussed what types of challenges would be good for him to take on. He increased his opportunities to go out alone, and by the 8th week, his FNE score had improved. After the 8th week, he became aware of the improvement in his symptoms, and he started going out to eat alone and making appointments to meet with others. In the second half of the sessions, he requested to practice interviewing for a job, and the interviewer conducted a mock job interview, along with free conversation. By the 14th week, he started job hunting; however, he had a very hurtful and depressing experience during the process and was unable to find a new job. His LSAS score improved during the 16th week, but his FNE score worsened. At the 24 week follow-up, he remained unemployed, and both his LSAS and FNE had worsened.

### Case 2

Case 2 was a 35 year-old female with a 20 year illness duration. Although she had been working, she wanted to change her job; she mentioned that she wanted to improve her condition so that she could complete a job interview. While she said she was not afraid of the therapist’s smiling and normal facial expressions, she was afraid of the sad face. Objectively, when the interviewer’s expression changed to sad, the patient’s facial expression stiffened and she had difficulty speaking. While engaging in free conversation, exposure to the sad face was started during the third session, and the duration of exposure was gradually increased. The contents of the free conversation were negative memories of the past, such as how she could not do as well as others, how she had made mistakes in important choices in her life, and what kind of bad luck she had suffered. The therapist tried to listen empathetically and to identify what she was doing well and give positive feedback. However, as if to deny such positive feedback, the communication became repetitive, with the patient insisting what she was not doing well. Even when the therapist tried to talk about topics that did not seem to be related to her symptoms, she talked about her own negative episodes that were related to the topics. She also had a fear of eating in front of others and tried to have a free conversation with the interviewer while eating together over a computer monitor. However, the patient reported that she was very fearful, especially when the interviewer told her how she was eating, and she became very concerned about how she was being looked at, with her fear instantly intensifying. The patient began to experience challenges in life situations outside of the sessions, such as communicating within the workplace, which she had been having difficulty with, and going out to eat with friends. She reported that her fear of the therapist’s sad expressions did not disappear until the last session. Her LSAS score gradually improved and the improvement had persisted at the 24 week follow-up, but her FNE score did not improve. In addition, the patient was unable to gain the confidence needed to interview for a new job.

### Case 3

Case 3 was a 21 year-old female with a 9 year illness duration. The patient’s baseline LSAS score was the lowest among the participants, but she expressed a strong fear of sad faces. At her request, she spent most of the interview time viewing the smiling face until the 10th session. Even viewing the smiling face, the patient was highly nervous and sometimes cried, although this gradually ceased during the sessions. At the beginning of the session, the conversation focused on the patient’s student life, past memories, and family, and the interviewer listened sympathetically as the patient sometimes talked about things related to her current medical history. The patient began to reflect more on things related to her symptoms and became aware that she was afraid that people might be angry with her. She also said that she had only vaguely recognized feelings of anxiety; however, she was now able to think objectively about why she was anxious and what effects anxiety had on her. From the 11th session, the patient began to choose a normal facial expression most of the time. When the sad face was attempted in two sessions, the patient rarely looked at the monitor and became grim. During the 12th session, the patient noticed that she was not good at asking questions about others during free conversation, and from the 13th session, she began to practice taking on different roles (e.g., she was a senior member of a club, and the interviewer was a junior member), asking questions, and trying to make the free conversation livelier. Both her LSAS and FNE scores improved, and the improvements had persisted at the follow-up.

## Discussion

Only Case 4, who showed lower L-SAS and FNE scores on the baseline than the other participants, dropped out of the study. While it is not entirely impossible that the dropout could have been caused by the interventions used in this study, there is a clear reason that a natural disaster made it difficult for Case 4 to continue the study, and we have determined that the software used in this study can be safely applied to the treatment of SAD. A previous study has reported a mean WAI-SF score of 77.9 for patients with anxiety disorders who underwent 16 weeks of video conference-delivered cognitive behavioral therapy intervention (Matsumoto et al., 2018). In addition, another study reported that psychiatric patients treated with telepsychiatry had a mean CSQ-8 score of 21.6 after 4 months (Bishop et al., 2002). Our results are comparable to these previous studies. The WAI-SF and CSQ-8 scores remained high throughout the study in all the cases, suggesting that this system does not affect therapeutic alliance or patient satisfaction. Working alliance is an important variable in psychotherapy (Sharf et al., 2010; Flückiger et al., 2018), and cognitive behavioral therapy for SAD has also shown that working alliance is related to treatment efficacy and study dropout (Haug et al., 2016). Although a few reports have examined how the use of VR affects the working alliance, previous studies of VRET for SAD have reported that VRET did not affect the working alliance, when compared with *in-vivo* exposure (Anderson et al., 2013; Bouchard et al., 2017). In VRET, high presence might reduce the tendency for patients to drop out of treatment, thereby increasing the effectiveness of the therapy (Robillard et al., 2003; Krijn et al., 2004; Price and Anderson, 2007). We thought the software we used is likely to achieve a higher presence than the use of VR animation because it displays the therapist’s face with real-time modifications. Future research should verify such things.

All participants reported that changing facial expressions also changed their feelings of fear, suggested that the software used in this study may be used to provide graded exposure therapy to SAD patients. In the future, we will need to confirm scientifically whether patient anxiety can be regulated. The ability to modulate patient anxiety may help to reduce the treatment burden, preventing dropout and increasing treatment satisfaction. In fact, Cases 2 and 3 were anxious about their usual medical visits, and they reported that conducting the interview using the system’s smiling face helped to reduce their anxiety. These findings suggest that a smiling face may facilitate the introduction of treatment for SAD.

Although this system required a 3-dimensional camera and a personal computer, it was easy to use and the modified facial expressions appeared natural without any discomfort. Therefore, the system can be easily applied to actual clinical practice. The use of telemedicine has recently become more widespread worldwide because of the COVID-19 pandemic (Kinoshita et al., 2020), and combining telemedicine with technology such as that used in this study may increase the likelihood of treatment for severe SAD patients, including those who are experiencing social withdrawal. Although the course of treatment varied from case to case, some cases showed notable improvement, despite the use of unstructured interviews, free conversations, and non-specific psychotherapy.

Cases 1 and 2 started their exposure to the sad face during the third session and increased the duration of exposure thereafter. Neither of them achieved their goal of getting new employment, but the changes that occurred throughout the sessions were different.

Case 1 was very positive about the study, and gradually became less fearful of sad faces; he seemed to gradually increase his self-efficacy as the sessions progressed. He was willing to increase what he could do outside of the sessions, and from the 5th session, he increased the range of his activities and seemed to become more confident. Unfortunately, his LSAS and FNE scores worsened after a job search that did not go well and a hurtful experience. Nevertheless, he seemed to have been improving steadily up until then.

In contrast, Case 2 continued to talk negatively about herself, asserting her lack of confidence until the end of the study. Her fear of the sad face did not decrease, and while her LSAS score decreased, her FNE score did not improve. Exposure therapy is a treatment method in which extinction learning occurs by experiencing anxious situations in a safe environment. Extinction learning is a new learning modulated by context, rather than erasing the original learning (Bouton, 2004). In Cases 1 and 2, the interviewer gave positive feedback with a sad face, which may have sounded sarcastic in this context, or the patient may have felt that the interviewer was giving a negative evaluation. Case 1 was convinced that the interviewer’s sad face was artificially created, and while he felt fear of the sad face, he also felt reassured by the positive evaluation. On the other hand, Case 2 stated that even though the interviewer was giving a positive evaluation, she felt that she was being evaluated negatively inwardly. Thus, she repeatedly denied the interviewer’s positive feedback. For Case 2, the positive feedback combined with a sad face led to her experiencing a feeling of being negatively evaluated as a result of talking about herself. Additionally, since the contents of the negative evaluation were unknown, she was unlikely to have any perception that it is safe to be evaluated negatively by the interviewer. Rather, she may have continued to have a vague fear that the evaluation might lead to something bad. Therefore, extinction learning for the FNE was unlikely to have occurred, and her score did not improve. Incidentally, the FNE has been considered a core cognitive bias that causes maladjustment in SAD patients (Weeks et al., 2005). However, in recent years, fear of receiving praise or positive feedback in social situations, that is fear of positive evaluation (FPE), has also come to be considered as an important cognitive component of social anxiety (Wallace and Alden, 1995; Fredrick and Luebbe, 2020). It is possible that the FPE score might have increased in Case 2, although FPE was not measured in this study.

Meanwhile, Case 3 appeared to be sufficiently fearful even when the smiling face was used; only the smiling face was used until the 10th session, and the sad face was rarely used. Nevertheless, she began to deepen her introspection about her medical condition, to think of ways to practice improving her communication skills, and thus to take control of her fear on her own. As a result, both her LSAS and FNE scores improved and the improvements persisted until the end of treatment. Reportedly, highly socially anxious individuals tend to avoid smiling even if they evaluate it positively (Heuer et al., 2007). Even subjects who are unaware of their fear of smiling may be unconsciously or biologically fearful of smiling; thus, smiling may be useful as an exposure stimulus. Furthermore, since SAD patients tend to be more concerned about what others think of them, it seems that even a smile can elicit fear, especially if the facial expression is not consistent with the context of the conversation. Consequently, even if the patient is aware that smiling does not elicit fear, as in Case 2, it may be worthwhile to continue smiling sessions for a longer period of time, while carefully monitoring the patient’s condition. Additionally, previous studies on communication with virtually transformed the facial expressions as used in our system have also shown that the use of smiling improves the smoothness of conversations during web conferencing, enhances creativity during collaborative work, and promotes idea generation (Nakazato et al., 2014; Suzuki et al., 2017). As with Case 3, it may also be effective when used for exposure therapy for SAD patients. Smiling may facilitate the generation of ideas for treatment and enhance working alliances by facilitating collaborative communication, which may ultimately lead to therapeutic benefits.

The present study was a pilot study and was limited to three cases. Since we did not have a therapeutically planned exposure group and there was no control group, whether the observed improvements in symptoms were due to our systems could not be determined. However, the accumulation of examples and further exploration of effective utilization methods may be worthwhile in the future.

## Conclusion

Interviews performed by modifying the therapist’s facial expressions via a web conferencing system could be conducted without causing treatment dropout or adverse events. During treatment for SAD, changing the therapist’s facial expression to a smile over the monitor may increase the effectiveness of the treatment by reducing resistance to the treatment and improving the therapeutic relationship. In addition, changing to a sad facial expression elicited fear in the SAD patients, suggesting that facial expression modification could be used as a graded exposure stimulus.

## Data availability statement

The datasets presented in this article are not readily available because our raw data contains personally identifiable information. Requests to access the datasets should be directed to TK, taishiro-k@mti.biglobe.ne.jp.

## Ethics statement

The studies involving human participants were reviewed and approved by the ethics committee of Shonan Keiiku Hospital. The patients/participants provided their written informed consent to participate in this study. Written informed consent was obtained from the individual(s) for the publication of any identifiable images or data included in this article.

## Author contributions

TH contributed to the design of the study, performed all the interventions, collected the data, and wrote the manuscript. SY and TT developed the system and provided technical assistance. MM and TK contributed to the design of the study and the writing of the manuscript. All authors contributed to the article and approved the submitted version.

## Funding

This study was funded by Novartis Pharma for a research grant. The funder was not involved in the study design, collection, analysis, interpretation of data, and the writing of this article or the decision to submit it for publication.

## Conflict of interest

SY is employed by OMRON SINIC X Corporation.

The remaining authors declare that the research was conducted in the absence of any commercial or financial relationships that could be construed as a potential conflict of interest.

## Publisher’s note

All claims expressed in this article are solely those of the authors and do not necessarily represent those of their affiliated organizations, or those of the publisher, the editors and the reviewers. Any product that may be evaluated in this article, or claim that may be made by its manufacturer, is not guaranteed or endorsed by the publisher.
